# Developmental Origin and Evolution of Bacteriocytes in the Aphid–Buchnera Symbiosis

**DOI:** 10.1371/journal.pbio.0000021

**Published:** 2003-10-13

**Authors:** Christian Braendle, Toru Miura, Ryan Bickel, Alexander W Shingleton, Srinivas Kambhampati, David L Stern

**Affiliations:** **1**Department of Ecology and Evolutionary Biology, Princeton UniversityPrinceton, New JerseyUnited States of America; **2**Laboratory for Development and Evolution, University Museum of ZoologyCambridgeUnited Kingdom; **3**Department of Biology, Graduate School of Arts and SciencesUniversity of Tokyo, TokyoJapan; **4**Department of Entomology, Kansas State UniversityManhattan, KansasUnited States of America

## Abstract

Symbiotic relationships between bacteria and insect hosts are common. Although the bacterial endosymbionts have been subjected to intense investigation, little is known of the host cells in which they reside, the bacteriocytes. We have studied the development and evolution of aphid bacteriocytes, the host cells that contain the endosymbiotic bacteria Buchnera aphidicola. We show that bacteriocytes of Acyrthosiphon pisum express several gene products (or their paralogues): Distal-less, Ultrabithorax/Abdominal-A, and Engrailed. Using these markers, we find that a subpopulation of the bacteriocytes is specified prior to the transmission of maternal bacteria to the embryo. In addition, we discovered that a second population of cells is recruited to the bacteriocyte fate later in development. We experimentally demonstrate that bacteriocyte induction and proliferation occur independently of B. aphidicola. Major features of bacteriocyte development, including the two-step recruitment of bacteriocytes, have been conserved in aphids for 80–150 million years. Furthermore, we have investigated two cases of evolutionary loss of bacterial symbionts: in one case, where novel extracellular, eukaryotic symbionts replaced the bacteria, the bacteriocyte is maintained; in another case, where symbionts are absent, the bacteriocytes are initiated but not maintained. The bacteriocyte represents an evolutionarily novel cell fate, which is developmentally determined independently of the bacteria. Three of five transcription factors we examined show novel expression patterns in bacteriocytes, suggesting that bacteriocytes may have evolved to express many additional transcription factors. The evolutionary transition to a symbiosis in which bacteria and an aphid cell form a functional unit, similar to the origin of plastids, has apparently involved extensive molecular adaptations on the part of the host cell.

## Abstract 2

Molecular markers show that bacteriocytes, the aphid cells that house the bacterial endosymbionts, are specified in a conserved two-step process that does not depend on the presence of the bacteria

## Introduction

Endosymbiosis is common in insects, with more than 10% of insect species relying upon intracellular bacteria for their development and survival (Baumann et al. 2000). Full genome sequencing of the endosymbiotic bacteria, Buchnera aphidicola, of several species of aphids has revealed extensive gene loss (Shigenobu et al. 2000; Tamas et al. 2002; van Ham et al. 2003), but has failed to reveal the genetic basis for the interaction between the bacteria and host cells. The key adaptations that allow incorporation of the bacteria into host cells may therefore be encoded by the host genome.

**Figure 1:**
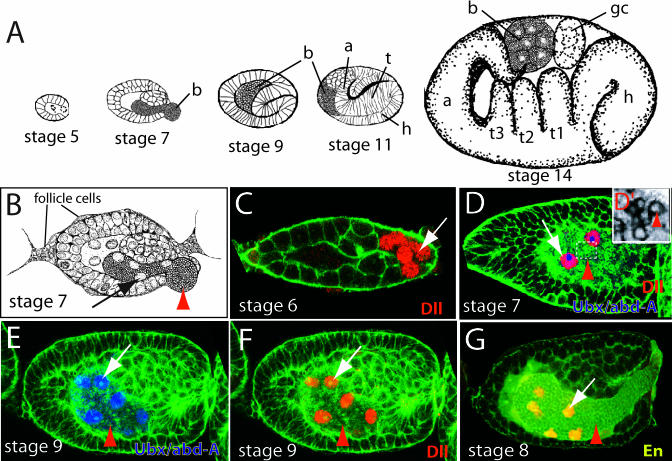
Expression of Three Transcription Factors during Early Bacteriocyte Development (A) Drawings of some stages of pea aphid embryonic development, approximately to scale. Embryos develop viviparously within a follicular epithelium of the ovariole (data not shown). For a complete description, see Miura et al. (2003). Bacteria are transferred at stage 7. Embryos are labeled with bacteria (b), head (h), thoracic (t), and abdominal (a) regions. The three thoracic segments (t1, t2, t2) and germ cells (gc) are indicated in the stage 14 embryo. (B) A drawing of a stage 7 embryo illustrates transovarial transfer of the bacteria (red arrowhead) to the embryo and the presumptive bacteriocyte nuclei (arrow). (C) Confocal micrograph of a stage 6 embryo stained with anti-Dll antibody (red, indicated by arrow). Anti-Dll labels syncytial nuclei (presumptive bacteriocyte nuclei) in the posterior of the embryo. (D) Confocal micrograph of stage 7 embryo stained with anti-Dll and FP6.87 antibodies. Soon after the bacteria begin to invade the embryo, we observe staining with the FP6.87 antibody localized to the nucleoli (blue), which recognizes both Ubx and Abd-A in diverse arthropods, in the same nuclei that are already expressing Dll (red). The region outlined with a broken white box is enlarged in (D′) to show the bacteria, and only the green channel is shown in monochrome. The red arrow indicates one bacterium. (E and F) In these two panels of the same focal plane from the same stage 9 embryo, Ubx/Abd-A staining (blue) is observed throughout the entire nucleus of all nuclei that also express Dll (red). (G) Confocal micrograph of a stage 8 embryo stained with anti-En (yellow). As the transfer of bacteria (arrowhead) is being completed, the bacteriocyte nuclei begin to express En (yellow, indicated with arrow). In (C)–(G), confocal micrographs show only one focal plane of the embryo, so not all bacteriocyte nuclei in each embryo can be seen. In all figures, F-actin is stained with phalloidin (green). Embryos in all figures, except Figure 2, are oriented with anterior of the entire embryo (towards the germarium) to the left.

The symbiotic bacteria of aphids, B. aphidicola, live within large polyploid cells, called bacteriocytes, that are grouped into organ-like structures, called bacteriomes, located adjacent to the ovarioles. During most of the aphid lifecycle, embryos develop parthenogenetically from unfertilized diploid oocytes, and multiple embryos develop serially within a single ovariole (Dixon 1985) (Figure 1A). Maternal bacteria are transferred directly to the developing blastoderm-stage embryos through an opening in the posterior of the embryo (Buchner 1965; Miura et al. 2003) (Figure 1B). Several researchers have described this transovarial transfer of bacteria (e.g., Uichanco 1924; Klevenhusen 1927; Toth 1933, 1938; Lampel 1958; Buchner 1965), but the details of bacteriocyte development have remained unclear.

We have identified bacteriocyte-specific markers that allow us to track the proliferation of bacteriocytes throughout the development of the pea aphid Acyrthosiphon pisum (Harris) (Hemiptera: Aphididae). Using these markers, we aimed to determine the developmental origin of bacteriocytes and to what extent bacteria are required for the formation of the bacteriocytes. We also tested whether the observed patterns of bacteriocyte development are evolutionarily conserved among distantly related aphid species. We show that three transcription factors are expressed in a specific temporal order during early bacteriocyte development of the pea aphid. The final population of bacteriocytes originates from two distinct populations of nuclei recruited at different times of development. Furthermore, we experimentally demonstrate that the specification and proliferation of bacteriocytes occur independently of B. aphidicola. In distant relatives of the pea aphid, we found that the two-step determination of bacteriocytes is conserved. We also investigated two cases involving the loss of B. aphidicola. In the first case, in which the bacterial symbionts have been replaced with extracellular, eukaryotic symbionts, bacteriocyte development appears to proceed normally. In a second case, in which males do not inherit B. aphidicola, the bacteriocytes have been lost.

## Results

### Three Transcription Factors Are Expressed in a Specific Temporal Order during Early Bacteriocyte Development

We tested five cross-reacting antibodies (see Materials and Methods) for their expression patterns in aphids. In every case, we observed antibody staining in the expected population of cells in the developing embryo (also see Miura et al. 2003). In addition, we found that three of the antibodies stained nuclei that form bacteriocytes of A. pisum. We infer that these antibodies are recognizing the homologues, or possibly paralogues, of their respective target proteins. The three proteins are expressed in a specific temporal order. We first observe expression of the Distal-less (Dll) protein (FlyBase ID: FBgn0000157) (Panganiban et al. 1994) in syncytial nuclei at the posterior of the blastoderm embryo just prior to the invasion of bacteria into the embryo (Figure 1C). As the bacteria enter the embryo, these nuclei associate with the bacteria and start to express a second protein, Ultrabithorax (Ubx) (FBgn0003944) or Abdominal-A (Abd-A) (FBgn0000014) or both, detected by the FP6.87 antibody (Kelsh et al. 1994) (Figure 1D–1F). The bacteria can be easily observed as spheres 2–4 μm in diameter (Buchner 1965) that stain with phalloidin (Figure 1D′). As the transfer of bacteria to the embryo is being completed, expression of the Engrailed (En) protein (FBgn0000577) (Patel et al. 1989) is detected (Figure 1G).

### Two Populations of Cells Are Recruited to the Bacteriocyte Fate at Different Times in Development

**Figure 2:**
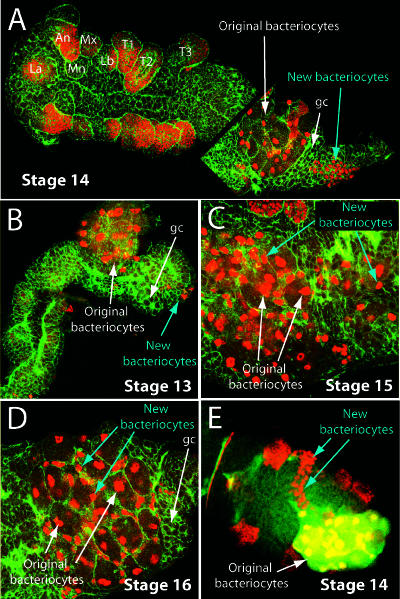
The Second Wave of Bacteriocyte Determination In (A)–(D), the embryos, which are normally folded in upon themselves in a pretzel shape within the ovariole (Miura et al. 2003), have been dissected flat, stained with anti-Dll antibody (red) and phalloidin (green), and examined with a confocal microscope. (A) Dll expression (red) in a stage 14 embryo is detected in the labrum (La) and all developing limbs on the ventral surface except the mandibular segment (Mn). (Other abbreviations: An, antenna; Mx, maxilla; Lb, labium; T1, T2, T3, first, second, and third thoracic leg, respectively.) The dorsal surface of the abdomen of the same embryo is shown illustrating Dll expression in the original bacteriocytes (white arrow) and in a more posterior population of nuclei or cells (blue arrow). Germ cells (gc) are labeled. (B) Dll expression is first observed in the new bacteriocyte nuclei at stage 13. (C) By stage 15, many of the new bacteriocytes have migrated to and begun intercalating between the original bacteriocytes. (D) By stage 16, all of the new bacteriocytes have intercalated between the original bacteriocytes. (E) The migration of the new bacteriocytes is seen in a confocal section of an undissected stage 14 embryo. Embryos in (A)–(D) are oriented with the anterior of the germband towards the left.

The early embryo contains approximately eight bacteriocyte nuclei that express Dll (Figure 1C), whereas the adult aphid contains 60–90 uninucleate polyploid bacteriocytes (Baumann et al. 2000) that also express Dll (data not shown). We found that the increase in bacteriocyte number occurs through two mechanisms. First, we infer that the original bacteriocyte nuclei divide, apparently in a syncytium and perhaps synchronously, through two rounds of division because we observe that the number of Dll-expressing nuclei increases from approximately eight to 16 by stage 12 and then to approximately 32 by stage 13 (data not shown). By stage 14, these original bacteriocytes have formed cell membranes and become polyploid (Figure 2A). At stage 13, a second population of approximately 40–60 cells located near the posterior end of the dorsal germband begins to express Dll (Figure 2B)*.* The nuclei of these cells are visibly smaller than those of the original bacteriocytes (Figure 2A–2E). Based on observations of multiple fixed specimens, we infer that these cells then migrate across the germband (Figure 2E) and intercalate between the original bacteriocytes (Figure 2C and 2D). The bacteria are presumably then subdivided among all of the Dll-expressing nuclei and the final bacteriocytes are formed.

### Bacteriocytes Are Specified and Maintained When the Bacteria Have Been Experimentally Removed

**Figure 3:**
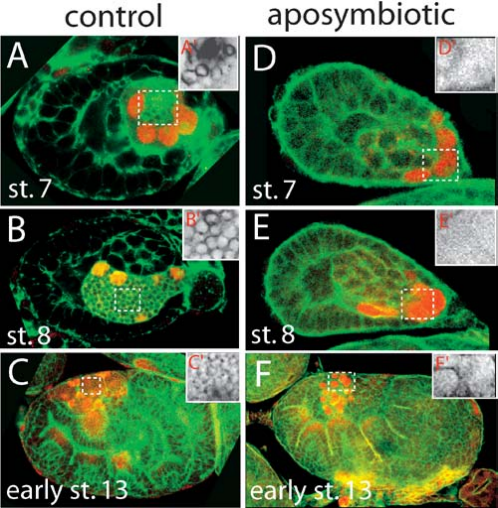
Elimination of B. aphidicola by Treatment with Antibiotics Has No Effect on the Determination and Maintenance of the Bacteriocyte Cell Fate in A. pisum (A–C) Confocal micrographs of control embryos stained with anti-Dll antibody (red) show expression of Dll, as described in Figure 1. Enlarged views of the bacteria within the broken white boxes in each embryo are shown in (A′)–(C′). (D–F) Embryos within aposymbiotic aphids at comparable stages as the controls in (A)–(C) express Dll in bacteriocyte nuclei. No bacteria are observed within these embryos, as seen in the enlarged views of (D′)–(F′).

The observations described in the first section suggest that the initial specification of the bacteriocyte may occur independently of B. aphidicola. We tested this idea by eliminating B. aphidicola from pea aphids by feeding aphids on an artificial diet containing antibiotics. We found that the embryos within these aposymbiotic aphids specify the bacteriocyte cell fate, as revealed by Dll expression, and maintain the bacteriocyte cell fate in the absence of bacteria (Figure 3). In addition, we have observed that the number of bacteriocytes in aposymbiotic embryos increases precisely as described for symbiotic embryos, including the second wave of bacteriocytes (Figure 3F; data not shown).

### The Two-Step Determination of Bacteriocytes Is Evolutionarily Conserved

**Figure 4:**
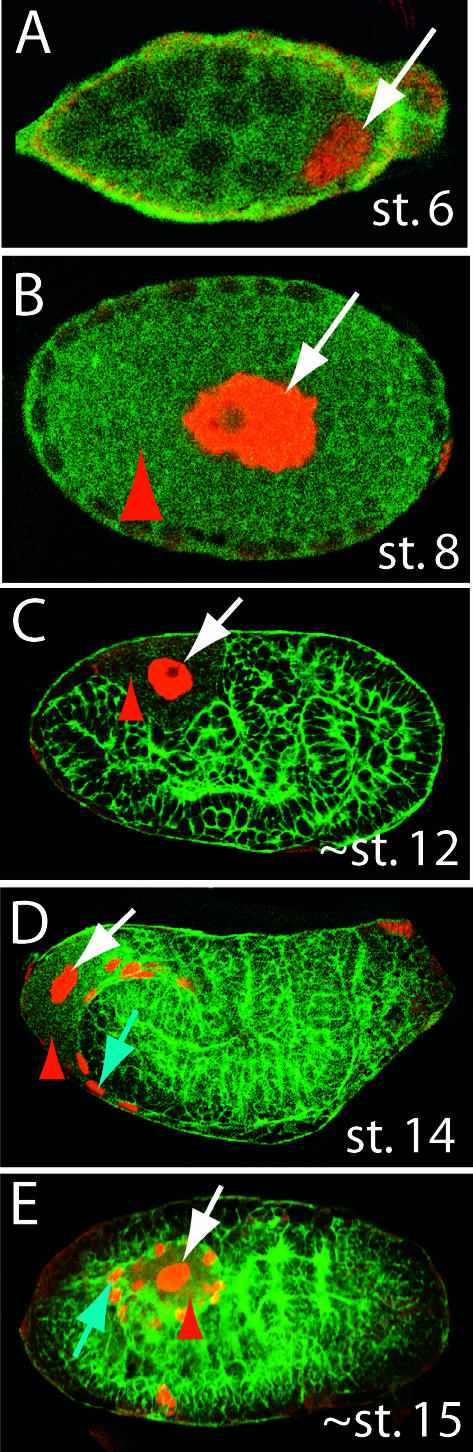
Expression of Dll in Bacteriocytes and the Pattern of Bacteriocyte Development Are Conserved in Parthenogenetic Females of P. spyrothecae Confocal micrographs of P. spyrothecae parthenogenetic embryos stained with anti-Dll antibody (red). (A) Dll is first detected in stage 6 embryos in one or two nuclei posterior to the cellular blastoderm (arrow). (B) By stage 8, the bacteria have been transferred to and entirely fill the embryo (red arrowhead). The Dll-expressing nuclei (arrow) have become highly polyploid. (C and D) At stage 12, only the original bacteriocyte nuclei are observed expressing Dll (white arrow), but by stage 14 (D) additional nuclei (blue arrow) closely apposed to the dorsal germband express Dll. (E) By stage 15, these new nuclei surround the original bacteriocyte, and at later stages the bacteria are divided into individual cells.

The two-step determination of bacteriocytes described in the previous sections appears to be a conserved feature of the aphids. Using the anti-Dll antibody, we examined development of the bacteriocytes in two species of aphids that diverged from A. pisum (subfamily Aphidinae) approximately 80–150 million years ago (von Dohlen and Moran 2000): Pemphigus spyrothecae (Eriosomatinae) and Tuberaphis styraci (Hormaphidinae) (discussed below). In both cases, Dll is expressed in a small number of bacteriocyte nuclei of the blastoderm-stage embryo and additional Dll-expressing cells are recruited later. In *P. spyrothecae,* one or two nuclei are originally determined as bacteriocytes, as suggested by Lampel (1958) (Figure 4A). These nuclei become highly polyploid prior to bacterial invasion and do not divide (Figure 4B and 4C). A second population of bacteriocytes is determined at approximately stage 14 (Figure 4D). These surround the original bacteriocyte (Figure 4E) and appear to divide the bacteria into independent bacteriocytes.

### Bacteriocytes Develop in Aphids in Which the Bacteria Have Been Replaced with Extracellular Eukaryotic Symbionts

**Figure 5:**
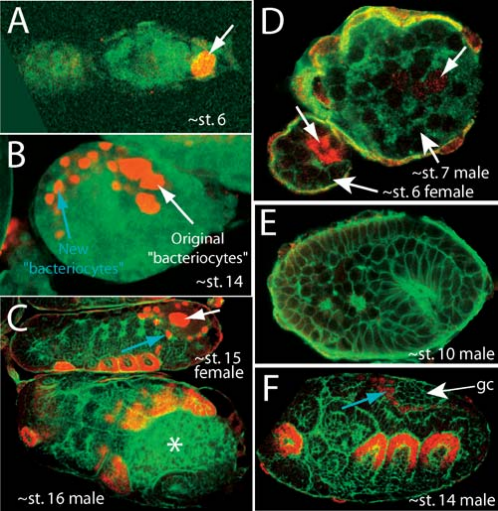
Bacteriocytes Are Retained in One Species That Has Evolutionarily Lost Bacteria, but Not in Males of Another Species That Do Not Inherit Bacteria (A and B) Confocal micrographs of embryos of T. styraci stained with anti-Dll antibody (red). In T. styraci, in which B. aphidicola has been evolutionarily lost (Fukatsu and Ishikawa 1992a), embryos still contain nuclei that express Dll in the correct time and place to be bacteriocyte nuclei. (A) Dll expression is first detected in posterior nuclei at blastoderm at approximately stage 6 (arrow). (B) By stage 14, the original nuclei have divided once or twice and become polyploid (original bacteriocytes), and new cells begin to express Dll (new bacteriocytes; blue arrow) and migrate towards the original bacteriocytes. (C–F) Confocal micrographs of embryos of P. spyrothecae stained with anti-Dll antibody (red). (C) Stage 16 male embryos of P. spyrothecae do not contain *B. aphidicola,* and no Dll-expressing cells are observed in the expected location for bacteriocytes. We believe that the cells in this location are sperm (marked with an asterisk). Sexual female embryos within the same ovary do contain Dll-expressing original and new bacteriocyte nuclei (white and blue arrows, respectively). (D and E) Transient expression of Dll in putative bacteriocytes is observed in stage 7 male embryos (arrow in male embryo of [D]), but this expression does not persist into stage 10 male embryos (E), where no Dll-expressing nuclei are observed. By contrast, stage 6 female embryos (D) contain polyploid Dll-expressing nuclei (arrow in female embryo of [D]). The sex of each embryo could be determined because males develop synchronously and earlier than females (Lampel 1958, 1968). (F) In stage 14 male embryos, we observe transient Dll expression in nuclei (blue arrow) adjacent to the germ cells (gc) in the correct location to be the second wave of bacteriocyte nuclei. This Dll expression does not persist (see stage 16 male in [C]), and the fate of the cells is unknown.

B. aphidicola ****has been lost in the lineage leading to T. styraci and has been replaced by a yeast-like symbiont (Buchner 1965; Fukatsu and Ishikawa 1992a; Fukatsu et al. 1994). These symbionts live in the hemolymph and occasionally invade cells of the fat body (Buchner 1965). Previous studies have therefore claimed that these species lack bacteriocytes (Buchner 1965; Fukatsu and Ishikawa 1992a). We found that these aphids contain one or two nuclei in the posterior of the blastoderm embryo that express Dll (Figure 5A). These nuclei divide once or twice and then become polyploid. At approximately stage 14, we observed a second population of Dll-expressing cells that migrate to the original Dll-expressing cells (Figure 5B). Therefore, T. styraci appears to retain the bacteriocyte cell fate although these cells do not apparently house the novel symbionts.

### The Bacteriocyte Fate Has Been Lost in Male Eriosomatine Aphids That Do Not Harbor B. aphidicola

Males of some species in the subfamily Eriosomatinae do not harbor B. aphidicola (Toth 1933; Buchner 1965; Fukatsu and Ishikawa 1992b). As these males lack mouthparts and do not feed, B. aphidicola are not required for growth. In addition, inheritance of B. aphidicola is strictly maternal, so males do not require symbionts for passage to their offspring. We did not detect any putative bacteriocyte cells that persist in male embryos of P. spyrothecae, although we observed them in female sexual embryos (Figure 5C and 5D). In stage 7 male embryos, we detected weak Dll expression in a few nuclei (Figure 5D), although this expression does not persist (Figure 5E). In addition, in stage 14 males we detected weak expression in cells that are in the correct location to be the second population of bacteriocytes (Figure 5F), but this expression also does not persist (see the stage 16 male in Figure 5C).

## Discussion

The aphid bacteriocyte expresses three transcription factors: Dll, En, and Ubx or Abd-A. These transcription factors play important roles during later stages of development in insects. For example, Dll is required for limb development, En is required for segmentation, and Ubx and Abd-A are the products of *Hox* genes, required for patterning thoracic and abdominal body regions (Kuner et al. 1985; Hidalgo 1996; Weatherbee et al. 1999; Panganiban and Rubenstein 2002). We know of no other cases in other insects in which any of these three transcription factors are expressed at such early stages of development as we have observed in the bacteriocytes (approximately cellular blastoderm). We cannot exclude the possibility that bacteriocytes evolved from a cell type that expressed this combination of transcription factors, but there are no obvious candidate cell types, such as fat cells or vitellophages, in other insects that fulfill this criterion. We do not yet know whether these genes are involved in the determination of bacteriocytes. However, bacteriocytes may require a novel combination of transcription factors to regulate the symbiont population and to mediate transovarial transmission.

We have demonstrated that two cell populations express Dll in spatially and temporally distinct patterns before incorporating bacteria. Our observation of the initial putative bacteriocytes in the blastoderm embryo is consistent with observations of earlier researchers, who suggested—based on morphological observations—that the nuclei located at the posterior of the embryo constitute the future bacteriocyte nuclei (Lampel 1958; Buchner 1965). In addition, we have found that the second population of presumptive bacteriocytes appears to migrate across the germband to the original bacteriocytes, where they take up bacteria. This is an unusual process that has not to our knowledge been described previously. In contrast, earlier studies indicated that bacteriocyte proliferation occurs solely by cell division or by budding of small nuclei from an existing polyploid bacteriocyte nucleus (e.g., Lampel 1958). We have not yet performed experiments that would allow us to positively identify the embryonic origin of this second population of cells. Based on their position—posterior to the germ cells and dorsal—these cells may be the descendants of the nuclei of the central syncytium (syncytial nuclei in the center of the blastoderm embryo) (see Miura et al. 2003).

Our results suggest that B. aphidicola is required for neither bacteriocyte induction nor for the origin and migration of the second population of bacteriocytes. While bacteria do not seem to be required for the developmental maintenance of this cell type, the bacteria may provide signals to the cells that are involved in mediating the symbiosis at the physiological level. Nonetheless, the absence of an effect of the bacteria on bacteriocyte development contrasts with other symbioses where the bacteria induce specific developmental changes in host tissues (McFall-Ngai and Ruby 1991).****

We investigated two cases in which B. aphidicola have been lost during the evolution of aphids. Given our observations that bacteria are not required for the developmental maintenance of bacteriocytes, it is possible that the bacteriocyte cell type might be lost if it had no other function. This does not appear to be the case. In the lineage including T. styraci, B. aphidicola was lost and a eukaryotic “yeast-like” symbiont has been gained (Buchner 1965; Fukatsu and Ishikawa 1992a; Fukatsu et al. 1994). Buchner (1965) suggested that the bacteriocytes of Cerataphis freycinetiae, another species in the same lineage, are originally specified, become polyploid and then degenerate. We found Dll-expressing putative bacteriocyte nuclei to be specified and maintained over extensive periods of embryonic development in T. styraci. Buchner documented considerable variation in the details of symbiotic transmission and bacteriocyte development, and it is possible that bacteriocyte development proceeds along different paths in these two species.

We also examined the development of bacteriocytes in males of P. spyrothecae. The males do not have bacteria and we have observed, consistent with observations of earlier researchers (Lampel 1958; Buchner 1965), that bacteriocytes are not maintained in this morph. We found that bacteriocytes initially express Dll, but this expression is not maintained, which is consistent with Lampel's and Buchner's observations that the original bacteriocytes appear to be present but are not maintained. In addition, we found that the second wave of bacteriocytes is also initiated, as shown by brief, weak Dll expression. It is not clear whether these cells are subsequently respecified or are eliminated.

B. aphidicola are derived from free-living bacteria (Baumann et al. 2000), and both the bacteriocyte and the symbiont must have evolved mechanisms for integrating the bacteria into the workings of the cell. The aphid–*Buchnera* symbiosis** represents a particularly intimate form of symbiosis. In some symbioses, the bacteria reside both intra- and intercellularly and actively invade the host cell (Dale et al. 2001). In contrast, B. aphidicola always exist either within host cells, within a membrane-bound maternal package, or with host nuclei in a syncytium. This advanced stage of symbiosis is similar to the presumptive early stages of plastid evolution.

## Materials and Methods

### 

#### Aphid rearing and collecting.

Colonies of A. pisum were reared on broad bean (Vicia faba) or alfalfa (Medicago sativa) (Miura et al. 2003). P. spyrothecae were collected from galls on Populus nigra var*. italica* in Cambridge and London, United Kingdom. T. styraci were collected from galls on Styrax obassia in Gunma Prefecture, Japan. Asexual aphid embryos of various developmental stages were dissected and fixed as described previously (Miura et al. 2003).

#### Antibody staining.

A limited number of antibodies recognize the homologues of their target proteins across insects. We tested five of these antibodies in aphids and found that three stained the bacteriocyte nuclei: rabbit anti-Dll (Panganiban et al. 1994), mouse anti-En (4D9) (Patel et al. 1989), and mouse anti-Ubx /Abd-A (FP.6.87) (Kelsh et al. 1994), kindly provided by G. Panganiban, N. Patel, and R. White, respectively. Two antibodies, rabbit anti-Vasa (FBgn0000606**)** (a gift of C.-C. Chang [Chang et al. 2002]) and mouse anti-Even-skipped (Eve) (2B8) (FBgn0003970) (Patel et al. 1994) did not stain bacteriocytes, but, as expected, anti-Vasa stained the germ cells (Chang et al. 2002) and anti-Eve stained cells in the nervous system (Patel et al. 1989). Secondary antibodies conjugated with fluorescent moieties (Jackson ImmunoResearch, West Grove, Pennsylvania, United States) were tested for cross-reactivity to aphid cells by staining embryos with secondary antibodies alone. No cross-reactivity was detected. We further tested whether an additional mouse antibody (mouse anti-digoxigenin; Jackson ImmunoResearch) cross-reacted with bacteriocyte nuclei, and it did not stain any parts of the aphid embryo. In addition, the anti-Dll, anti-En, and anti-Ubx/Abd-A all stained the expected cells (Patel et al. 1989; Kelsh et al. 1994; Panganiban et al. 1994; Miura et al. 2003) in other regions of the embryos, indicating that the antibodies were working as expected. Cell outlines were visualized by staining for F-actin with fluorescein-conjugated phalloidin. Embryos were stained using standard protocols (Miura et al. 2003) and visualized on Leica SP and Zeiss confocal microscopes.

#### Antibiotic treatment.

In the pea aphid, *Buchnera* can be eliminated by treating animals with antibiotics (Wilkinson 1998). First- or second-instar aphids were fed on an artificial diet containing 50 μg/ml of the antibiotic rifampicin for 72 h (e.g., Caillaud and Rahbé 1999). Aphids were then transferred to leaves of Medicago arborea in Petri-dish cultures (Miura et al. 2003). Control aphids were treated identically, except that the antibiotic was omitted from the artificial diet. Embryos that were less than 4 d old (Miura et al. 2003) were dissected from aposymbiotic aphids within 2–4 d after the end of the antibiotic treatment and stained with anti-Dll and FP6.87 antibodies and fluorescein-conjugated phalloidin. The absence of bacteria in aposymbiotic aphids was confirmed by observation with a confocal microscope (see Figure 3).

## Supporting Information

### 

#### Accession Numbers

The FlyBase accession numbers discussed in this paper are FBgn0000014, FBgn0000157, FBgn0000577, FBgn0000606, FBgn0003944, and FBgn0003970.

## Abbreviations

Abd-A: Abdominal-A
Dll: Distal-less
En: Engrailed
Eve: Even-skipped
Hox: homeotic complex
Ubx: Ultrabithorax.
